# *KRAS* mutation is a weak, but valid predictor for poor prognosis and treatment outcomes in NSCLC: A meta-analysis of 41 studies

**DOI:** 10.18632/oncotarget.7080

**Published:** 2016-01-30

**Authors:** Wei Pan, Yan Yang, Hongcheng Zhu, Youcheng Zhang, Rongping Zhou, Xinchen Sun

**Affiliations:** ^1^ Department of Radiation Oncology, The First Affiliated Hospital of Nanjing Medical University, Nanjing, Jiangsu, P.R. China; ^2^ Department of Oncology, The Second Hospital of Nanjing Jiangning, Nanjing, Jiangsu, P.R. China; ^3^ Department of Oncology, Nanjing Jiangning Hospital, Nanjing, Jiangsu, P.R. China

**Keywords:** non-small cell lung cancer, *KRAS* mutation, prognosis, EGFR-TKI, meta-analysis

## Abstract

Mutation of oncogene *KRAS* is common in non-small cell lung cancer (NSCLC), however, its clinical significance is still controversial. Independent studies evaluating its prognostic and predictive value usually drew inconsistent conclusions. Hence, We performed a meta-analysis with 41 relative publications, retrieved from multi-databases, to reconcile these controversial results and to give an overall impression of *KRAS* mutation in NSCLC. According to our findings, *KRAS* mutation was significantly associated with worse overall survival (OS) and disease-free survival (DFS) in early stage resected NSCLC (hazard ratio or HR=1.56 and 1.57, 95% CI 1.39-1.76 and 1.17-2.09 respectively), and with inferior outcomes of epidermal growth factor receptor-tyrosine kinase inhibitors (EGFR-TKIs) treatment and chemotherapy (relative risk or RR=0.21 and 0.66 for objective response rate or ORR, 95% CI 0.12-0.39 and 0.54-0.81 respectively; HR=1.46 and 1.30 for progression-free survival or PFS, 95%CI 1.23-1.74 and 1.14-1.50 respectively) in advanced NSCLC. When *EGFR* mutant patients were excluded, *KRAS* mutation was still significantly associated with worse OS and PFS of EGFR-TKIs (HR=1.40 and 1.35, 95 % CI 1.21-1.61 and 1.11-1.64). Although *KRAS* mutant patients presented worse DFS and PFS of chemotherapy (HR=1.33 and 1.11, 95% CI 0.97-1.84 and 0.95-1.30), and lower response rate to EGFR-TKIs or chemotherapy (RR=0.55 and 0.88, 95 % CI 0.27-1.11 and 0.76-1.02), statistical differences were not met. In conclusion, *KRAS* mutation is a weak, but valid predictor for poor prognosis and treatment outcomes in NSCLC. There's a need for developing target therapies for *KRAS* mutant lung cancer and other tumors.

## INTRODUCTION

Lung cancer, with NSCLC accounts for 85% of all cases, is the most common human malignant disease and the leading cause of cancer-related mortality worldwide [1, 2]. Early as the beginning of this century, novel molecular targeted agents like EGFR-TKIs represented by gefitinib or erlotinib, which interfere with EGFR signaling, have been proved dramatically effective for selected advanced NSCLC patients with sensitive *EGFR* mutations [3]. Since then, molecular target therapies provided promising treatment alternatives to surgery, radiation therapy and chemotherapy. Personalized, genotype-directed therapy for NSCLC couldn't be more popular. Besides *EGFR*, *KRAS* is the most frequently mutated oncogene in NSCLC (15-20%) with most cases affect exon 2 and 3 (G12, G13 and Q61). It seemed that *KRAS* mutation occurs more frequently in lung adenocarcinomas (approximately 30%), in the Caucasian population, and in the population with smoking history [4–6].

*KRAS* mutation was described as a negative prognostic marker for OS and DFS in lung adenocarcinoma more early in 1990 [7]. Not until the last ten years, clinical significance of *KRAS* mutation in NSCLC has been attracted more and more attention. Although a lot of published studies reported that *KRAS* mutation is associated with poor prognosis and outcomes of EGFR-TKIs treatment [8–11] and chemotherapy [10, 12–15], more than a few independent studies argued that it predicts neither worse prognosis [8, 10–12, 14, 16–28] nor inferior outcomes of EGFR-TKIs treatment or chemotherapy [14, 18, 19, 29–32]. Therefore, we carried out a comprehensively search and review of relevant publications in multi-database. Useful data was extracted and then aggregated by using a meta-analysis methodology to give an overall impression of *KRAS* mutation in NSCLC.

Moreover, it is accepted that sensitive *EGFR* mutation predicts benefit from EGFR-TKIs treatment and even from chemotherapy in NSCLC [8, 9, 18, 19, 26, 32-34]. Mutations of *KRAS* and *EGFR* are common and mutually exclusive in NSCLC [35–37]. Thus *EGFR* mutation predominantly coexists with wild type *KRAS*, which made us overestimate the prognostic and predictive value of *KRAS* mutation. Therefore, analyses were re-performed in *EGFR* wild-type NSCLC to obtain objective and unassertive conclusions.

## RESULTS

### Study characteristics and quality assessment

**Figure 1:**
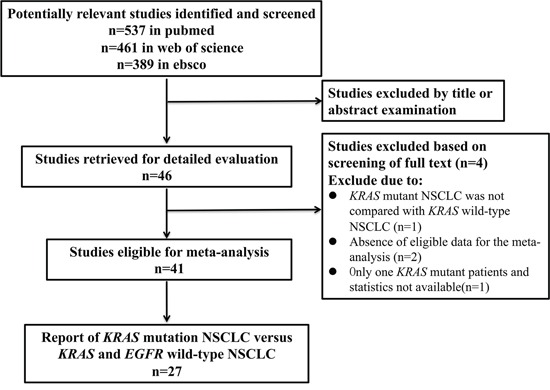
Flow Chart of publication search and selection

Based on our search criteria, a total of 41 studies, which enrolled 13,103 *KRAS* assessable patients with 18 percent (2,374) *KRAS* mutant positive cases, were eligible for inclusion in the present analyses. The process of selecting publications was presented in Figure 1 and the clinical characteristics of the included studies were listed in Table 1. All of the studies were published from 2005-2015, consisting of 40 cohort studies [8–31, 34, 35, 38-51] and one randomized controlled trial (RCT) [32]. Thirty studies [8, 9, 11–19, 21–28, 31, 35, 38, 40–43, 46–48, 51] conducted in Europe and North America, ten studies [10, 20, 29, 30, 32, 34, 39, 44, 45, 50] in Asia, and one study [49] in Latin America. All of the studies focused on NSCLC or lung adenocarcinoma only except one [46] on lung squamous cell carcinoma. Ten studies [16, 20, 24, 25, 29, 30, 39, 43, 47, 48] dealt with stage I-IIIa resected tumors, twenty-nine studies [8, 9, 11–15, 17–19, 21–23, 26–28, 31, 34, 35, 38, 40–42, 44–46, 49–51] with stage IIIb-IV unresectable tumors, and two studies [10, 32] with all stage tumors. Thirteen studies [10, 12, 13, 18, 29, 30, 34, 35, 41, 44, 46, 47, 49] used a polymerase chain reaction (PCR) or modified PCR method to test gene mutation, while the others used a direct sequencing method. Four studies [12, 13, 38, 45] assessed *KRAS* mutation in plasma DNA and the others in tumor specimens. In consistent with large-scaled demographic results [6], the majority of *KRAS* mutation occurs in codon 12, with G12C the most, occasionally in codon 13, and rarely in codon 61. All the studies selected patients randomly without concerning gender or smoking status and most results were adjusted for gender, age, stage and Karnofsky performance score.

**Table 1:** Clinical characteristics of included studies

First Author	Year	Race	Patients Number	*KRAS* MUT Number(%)	Gene Testing Method	Specimens Assessed	Pathology	Stage	Treatments	Outcome	*KRAS* MUT Versus	Quality Score
**William [35]**	2005	Caucasian	60	9 (15.0)	PCR	Tumor	ADC	IIIB-IV	TKI	ORR	WT/WT	6
**David [8]**	2005	Caucasian	274	55 (20.0)	Sequencing	Tumor	NSCLC	IIIB-IV	TKI / CT	OS / ORR / PFS	WT/WT	8
**Erminia [9]**	2007	Caucasian	70	16 (22.9)	Sequencing	Tumor	NSCLC	IIIB-IV	TKI	ORR / PFS	WT/WT	8
**Oliver [38]**	2007	Caucasian	175	16 (9.1)	Sequencing	Plasma	NSCLC	I-IV	Various	OS	WT	6
**Young [29]**	2008	Asian	71	5 (7.0)	PCR	Tumor	ADC	I-III	R	OS / DFS	WT	6
**Jenifer [16]**	2008	Caucasian	296	50 (16.9)	Sequencing	Tumor	ADC	I-III	R	OS	WT/WT	9
Chang-qi **[17]**	2008	Caucasian	206	30 (14.6)	Sequencing	Tumor	NSCLC	IIIB-IV	TKI	OS / ORR	NA	9
**Takayuki [39]**	2009	Asian	254	32 (12.6)	Sequencing	Tumor	ADC	I-III	R	OS	WT/WT	9
**MILOS [18]**	2009	Caucasian	208	32 (15.4)	PCR	Tumor	NSCLC	IIIB-IV	TKI	OS / PFS	WT/WT	7
**David [19]**	2009	Caucasian	175	41 (23.4)	Sequencing	Tumor	NSCLC	IIIB-IV	TKI	OS / ORR / PFS	WT/WT	8
**Tetsukan [30]**	2009	Asian	168	24 (14.3)	PCR	Tumor	ADC	I	R	DFS	WT	9
**Antonio [40]**	2009	Caucasian	83	16 (19.3)	Sequencing	Tumor	NSCLC	IIIB-IV	TKI	OS / ORR / PFS	WT/WT	7
Hui-ping **[20]**	2010	Asian	156	7 (4.5)	Sequencing	Tumor	NSCLC	I-III	R	OS	WT/WT	7
**Laura [41]**	2010	Caucasian	62	12 (19.4)	PCR	Tumor	ADC	IIIB-IV	TKI	OS / PFS	WT	8
**Vienna [21]**	2011	Caucasian	161	11 (6.8)	Sequencing	Tumor	NSCLC	IIIB-IV	TKI	OS / ORR / PFS	WT/WT	8
**Carlos [12]**	2011	Caucasian	308	27 (8.8)	PCR	Plasma	NSCLC	IIIB-IV	CT	OS / PFS	WT	8
**Hye [10]**	2011	Asian	229	19 (8.3)	PCR	Tumor	NSCLC	I-IV	R / CT / TKI	OS / DFS / ORR / PFS	WT/WT	6
**Wolfram [22]**	2011	Caucasian	493	90 (18.3)	Sequencing	Tumor	NSCLC	IIIB-IV	TKI	OS / PFS	WT/WT	9
**Vienna [42]**	2012	Caucasian	162	11 (6.8)	Sequencing	Tumor	NSCLC	IIIB-IV	TKI	OS / ORR / PFS	WT/WT	9
**Chiara [43]**	2012	Caucasian	249	46	Sequencing	Tumor	NSCLC	I-III	R	DFS	WT/WT	9
**Melissa [23]**	2012	Caucasian	1036	241 (23.3)	Sequencing	Tumor	ADC	IV	CT / TKI	OS	WT/WT	9
**Jie [44]**	2012	Asian	104	9 (8.7)	PCR	Tumor	NSCLC	IIIB-IV	Various	OS	WT	6
**Giulio [11]**	2012	Caucasian	67	18 (26.9)	Sequencing	Tumor	ADC	IIIB-IV	TKI	OS / ORR / PFS	WT/WT	7
**Jacques [31]**	2012	Caucasian	307	42 (13.7)	Sequencing	Tumor	NSCLC	IIIB-IV	TKI	OS / PFS	WT/WT	7
**Seung [45]**	2013	Asian	57	14 (24.6)	Sequencing	Plasma	NSCLC	IIIB-IV	TKI / BSC	OS / ORR	WT	8
**Anneli [13]**	2013	Caucasian	246	43 (17.5)	PCR	Plasma	NSCLC	IIIB-IV	CT	OS / ORR / PFS	WT	8
**Ondrej [46]**	2013	Caucasian	215	16 (7.4)	PCR	Tumor	SCC	IIIB-IV	TKI	OS / PFS	WT	7
Ji-lin **[32]**	2013	Asian	1935	98 (5.1)	Sequencing	Tumor	ADC	I-IV	R / TKI / CT	OS / DFS / ORR / PFS	WT/WT	3*
Jong-Mu **[34]**	2013	Asian	484	39 (8.1)	PCR	Tumor	ADC	IIIB-IV	TKI / CT	OS / ORR / PFS	WT/WT	9
**Frances [25]**	2013	Caucasian	1543	300 (19.4)	Sequencing	Tumor	NSCLC	I-III	R	OS / DFS	WT	9
**Gerald [26]**	2013	Caucasian	368	110 (29.9)	Sequencing	Tumor	NSCLC	IIIB-IV	TKI	OS	WT/WT	6
**Wouter [14]**	2013	Caucasian	161	60 (37.3)	Sequencing	Tumor	ADC	IIIB-IV	CT	OS / ORR / PFS	WT	8
**Giulio [15]**	2014	Caucasian	204	77 (37.7)	Sequencing	Tumor	ADC	IIIB-IV	CT	OS / ORR / PFS	WT/WT	9
**Marianna [27]**	2014	Caucasian	108	39 (36.1)	Sequencing	Tumor	NSCLC	IIIB-IV	CT	OS / ORR / PFS	WT/WT	9
**Mihaly [28]**	2014	Caucasian	1125	361 (32.1)	Sequencing	Tumor	ADC	IIIB-IV	CT	OS / ORR / PFS	WT/WT	9
**Mark [24]**	2014	Caucasian	230	39 (17.0)	Sequencing	Tumor	ADC	I-III	R	OS / DFS	WT/WT	8
**Benjamin [47]**	2014	Caucasian	312	127 (40.7)	PCR	Tumor	ADC	I	R	OS / DFS	WT/WT	8
**Ernest [48]**	2015	Cacasian	179	85 (47.5)	Sequencing	Tumor	ADC	I-III	R	OS / DFS	WT	8
**Alma [49]**	2015	Other	225	40 (17.8)	PCR	Tumor	NSCLC	IIIB-IV	TKI / CT	OS / ORR / PFS	WT	8
**Shigehiro [50]**	2015	Asian	119	16 (13.4)	Sequencing	Tumor	ADC	IIIB-IV	CT	OS / ORR / PFS	WT/WT	8
**Eliana [51]**	2015	Caucasian	218	51 (23.4)	Sequencing	Tumor	NSCLC	IIIB-IV	TKI / CT	OS / ORR / PFS	WT/WT	8

**MUT**, mutation; **NSCLC**, non-small cell lung cancer; **ADC** lung adenocarcinoma; **SCC**, lung squamous cell carcinoma; **CT**, chemotherapy; **R**, surgical resection; **WT**, *KRAS* wild-type; **WT/WT**, *KRAS* and *EGFR* wild-type.

* randomized controlled trial was evaluated based on Jadad Scale.

The quality of cohort study was assessed using the Newcastle-Ottawa Scale (NOS) on three perspectives: patient selection, comparability of groups, and assessment of outcome. Full score is nine stars, and a study with more stars was considered to be of higher quality. Quality scores of 40 cohort studies ranged from six to nine with a median score of eight. The quality of RCT was assessed using the Jadad Scale on three perspectives: randomization, double blinding, withdraws and dropouts. Full score is five points, and a study with score no less than three points is defined the high-quality study. The only included RCT gained a score of three points. No “poor quality” study was found and all of the studies were considered acceptable for inclusion in the present meta-analysis. The study specific scores were summarized in Table S1.

### *KRAS* mutation and clinical features

Data of clinical features stratified by *KRAS* mutational status was reported in 25 studies [9, 10, 12–17, 21, 23, 24, 26–28, 30, 32, 34, 39, 41, 44–48, 50]. Data was extracted from individual studies and then aggregated. The result indicated that *KRAS* mutation occurs more frequently in lung adenocarcinoma (RR=1.16 p=0.016), and in former or current smokers (RR=1.13 p=0.017), but not in male gender (RR=1.07 p=0.142) (Table S2). Reported gene mutation rate ranged from 4.4% to 24.5% in the Asians and from 6.7% to 47.4% in the Caucasians. Additionally, an increased incidence of presence of stage IV disease and distant metastasis in *KRAS* mutant patients was reported in several studies [27, 50].

### Prognostic and predictive value of *KRAS* mutation in unselected NSCLC

**Figure 2:**
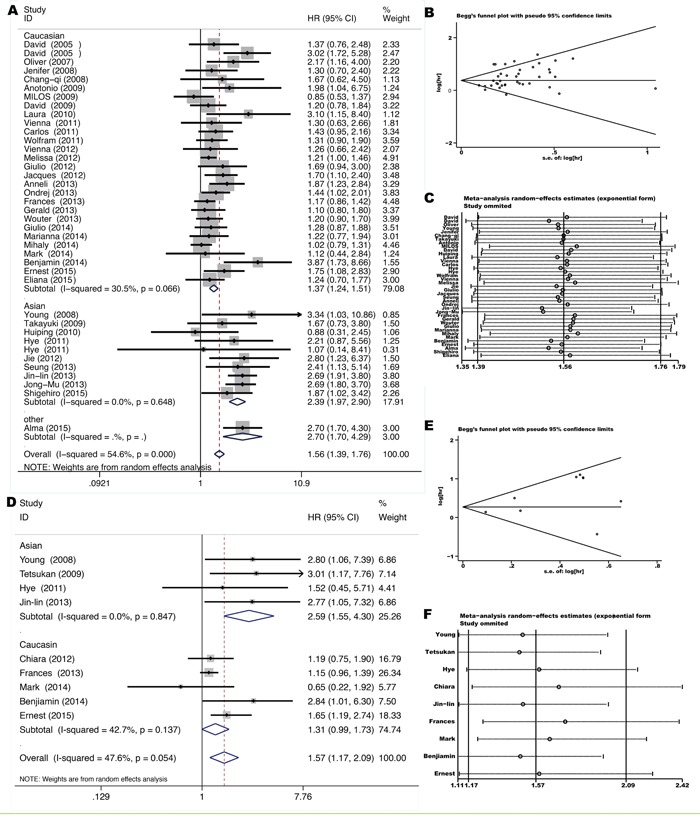
Forrest plot **A, D.** with influence analysis **C, F.** of hazard ratio for overall survival and disease-free-survival comparing *KRAS* mutant patients with *KRAS* wild-type patients. Begg's funnel plot of enrolled studies for estimating the hazard ratio for overall survival **B.** and disease-free-survival **E.**

Thirty-seven studies [8, 10–29, 31, 32, 34, 38–42, 44–51] provided HRs for OS comparing *KRAS* mutant NSCLC with *KRAS* wild-type NSCLC. Pooled HR was 1.56 for OS (95%CI 1.39-1.76, p=0.00) (Figure 2A), indicating a significantly worse survival for *KRAS* mutant patients. Significant heterogeneity among studies (*I^2^* =54.6%, p=0.00) and publication bias (Begg's test p=0.053, Egger's test p=0.014) (Figure 2B) was detected. Meta-regression analysis showed that only race (adjusted R^2^=77.12%, p=0.00) might contribute to the heterogeneity, but not other factors such as disease stage (p=0.885), pathology (p=0.454), gene mutation testing method (p=0.029) and specimens (plasma/tumor foci) for mutation assessment (p=0.560). As shown in Figure 2A, subgroup analysis according to race showed that *KRAS* mutation is a more powerful negative prognostic factor for OS in the Asians (HR=2.39 with 95% CI 1.97-2.90 and p=0.00, *I^2^*=0.0% and p=0.648 for heterogeneity) than in the Caucasians (HR=1.37 with 95%CI 1.24-1.51 and p=0.00, *I^2^*=30.5 and p=0.066 for heterogeneity).

Nine studies [10, 24, 25, 29, 30, 32, 43, 47, 48] dealt with stage I-IIIa resected NSCLC and provided HRs for DFS comparing *KRAS* mutant tumors with *KRAS* wild-type tumors. All cases received R0 resection and lobectomies were performed mostly. Pooled HR was 1.57 for DFS (95% CI 1.17-2.09, p=0.002) (Figure 2D), indicating an increased hazard for disease recurrence after tumor resection for *KRAS* mutant patients. Neither significant heterogeneity (*I^2^* =47.6%, p=0.054) nor publication bias (Begg's test p=0.754, Egger's test p=0.062) (Figure 2E) was detected. However, meta-regression analysis showed that significant heterogeneity did exist between two races (adjusted R^2^=−85.65%, p=0.042). Similarly, subgroup analysis according to race showed that *KRAS* mutation is a more powerful negative prognostic factor for DFS in the Asians (HR=2.59, 95% CI 1.55-4.30 and p=0.00, *I*^2^=0.0% and *p*=0.847 for heterogeneity) than in the Caucasians (HR=1.31, 95% CI 0.99-1.73 and p=0.057, *I*^2^=42.7% and *p*=0.137 for heterogeneity) (Figure 2D).

**Figure 3:**
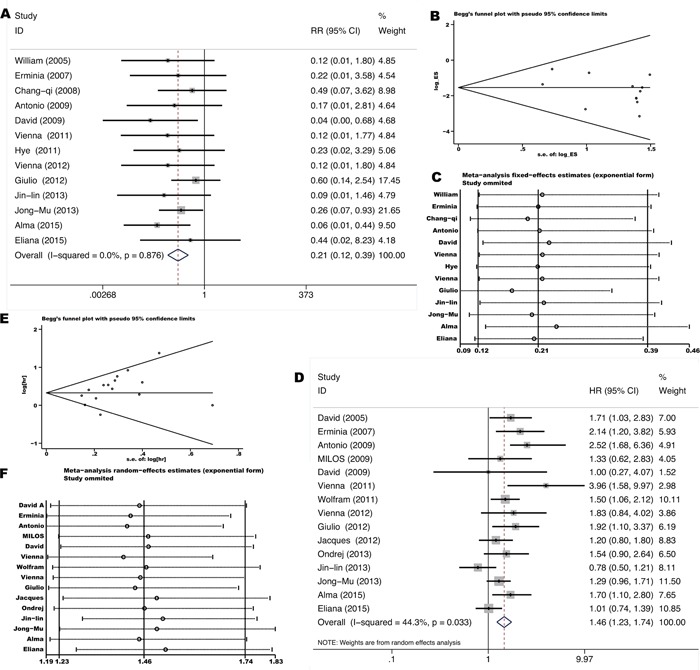
Forrest plot of relative ratio for objective response rate **A.** and hazard ratio for progression-free-survival **D.** with influence analysis **C, F.** comparing *KRAS* mutant patients with *KRAS* wild-type patients treated with EGFR TKIs. Begg's funnel plot of enrolled studies for estimating the relative ration for overall response **B.** and hazard ratio for progression-free-survival **E.**

Eighteen studies [9–11, 17–19, 21, 22, 31, 32, 34, 35, 40–42, 46, 49, 51] investigated outcomes (response rate or PFS) of EGFR-TKIs treatment in stage IIIb-IV unresectable NSCLC comparing *KRAS* mutant tumors with *KRAS* wild-type tumors. Either gefitinib or erlotinib was administered in standard dosage as first to three-line treatment. The total ORR (complete response or CR + partial response or PR) was 2.5% (6/237) in *KRAS* mutant patients and 34.0% (499/1469) in *KRAS* wild-type patients. Pooled RR was 0.21 for ORR (95% CI 0.12-0.39, p=0.00) (Figure 3A) while pooled HR was 1.46 for PFS (95% CI 1.23-1.74, p=0.0) (Figure 3D), indicating a significant lower response rate and shorter remission period of EGFR-TKIs treatment for *KRAS* mutant patients. Neither significant heterogeneity (*I^2^*=0.0%, p=0.876 and *I^2^* =44.3%, p=0.033 respectively) nor publication bias (Begg's test p=0.502, Egger's test p=0.086 and Begg's test p=0.06, Egger's test p=0.053 respectively) (Figure 3B and 3E) was detected. Meta-regression analysis showed that neither race (p=0.440) nor gene mutation testing method (p=0.807) contributes significantly to the heterogeneity.

**Figure 4:**
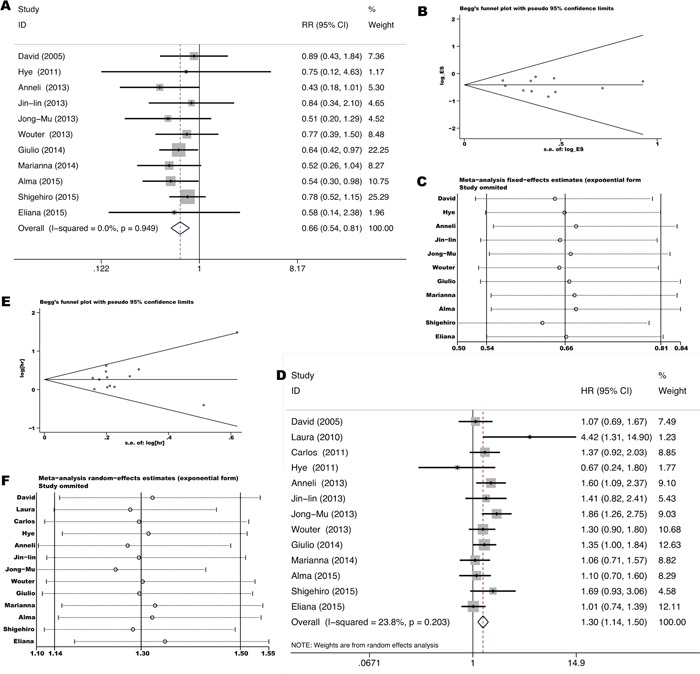
Forrest plot of relative ratio for objective response rate **A.** and hazard ratio for progression-free-survival **D.** with influence analysis **C, F.** comparing *KRAS* mutant patients with *KRAS* wild-type patients treated with chemotherapy. Begg's funnel plot of enrolled studies for estimating the relative ration for overall response **B.** and hazard ratio for progression-free-survival **E.**

Thirteen studies [8, 10, 12–15, 27, 32, 34, 41, 49–51] investigated outcomes of chemotherapy in stage IIIb-IV unresectable NSCLC comparing *KRAS* mutant tumors with *KRAS* wild-type tumors. Platinum-based doublet was used for first to second-line treatment. The total ORR was 21.1% (82/389) in *KRAS* mutant patients and 32.9% (486/1477) in *KRAS* wild-type patients. Pooled RR was 0.66 for ORR (95% CI 0.54-0.81, p=0.00) (Figure 4A) while pooled HR was 1.30 for PFS (95% CI 1.14-1.50, p=0.0) (Figure 4D), indicating a significant lower response and shorter remission period of chemotherapy for *KRAS* mutant patients. Neither significant heterogeneity (*I^2^*=0.0%, p=0.949 and *I^2^* =23.8%, p=0.203 respectively) nor publication bias (Begg's test p=0.755, Egger's test p=0.506 and Begg's test p=0.583, Egger's test p=0.419 respectively) (Figure 4B and 4E) was detected. Meta-regression analysis showed that neither race (p=0.736) nor gene mutation testing method (p=0.389) contributes significantly to the heterogeneity.

### Prognostic and predictive value of *KRAS* mutation in *EGFR* wild-type NSCLC

Additionally, twenty-seven studies [8–11, 15, 16, 18–24, 26–28, 31, 32, 34, 35, 39, 40, 42, 43, 47, 50, 51] including 9,383 both *KRAS* and *EGFR* assessable patients investigated the prognostic and predictive value of *KRAS* mutation in *EGFR* wild-type NSCLC. Although mutations of *KRAS* and *EGFR* were mutually exclusive in most cases [35–37], presence of both gene mutations could be seen occasionally [18, 34, 47].

**Figure 5:**
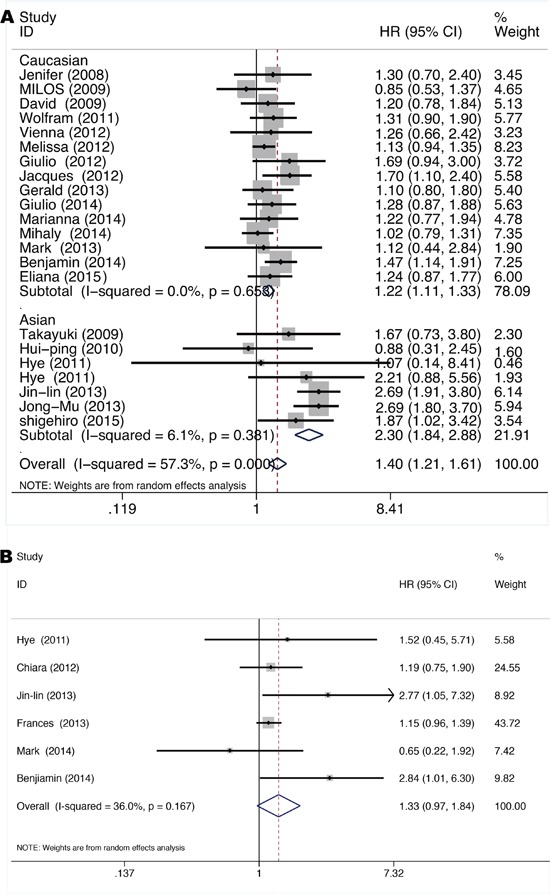
Forrest plot of hazard ratio for overall survival **A.** and disease-free-survival **B.** comparing *KRAS* mutant patients with *KRAS* and EGFR wild-type patients.

As shown in Figure 5A, pooled HR was 1.40 for OS (95% CI 1.21-1.61, p=0.0) based on 21 studies [10, 11, 15, 16, 18–20, 22–24, 26–28, 31, 32, 34, 39, 42, 47, 50, 51] comparing *KRAS* mutant NSCLC with *KRAS* and *EGFR* wild-type NSCLC, indicating a significant worse survival for *KRAS* mutant patients. Significant heterogeneity among studies (*I^2^* =57.3%, p=0.0) but not publication bias (Begg's test p=0.866, Egger's test p=0.486) (Figure S1A) was detected. Similarly, meta-regression analysis showed that only races (adjusted R^2^=95.14%, p=0.0) might contribute to the heterogeneity. Subgroup analysis according to races showed that *KRAS* mutation impairs survival more seriously in the Asians (HR=2.30 with 95% CI 1.84-2.88 and p=0.0, *I^2^*=6.1% and p=0.381 for heterogeneity) than in the Caucasians (HR=1.22 with 95% CI 1.11-1.33 and p=0.00, *I^2^*=0.0% and p=0.653 for heterogeneity) (Figure 5A).

As shown in Figure 5B, pooled HR was 1.33 for DFS (95% CI 0.97-1.84, p=0.076) based on six studies [10, 24, 25, 32, 43, 47] conducted in stage I-IIIa resected NSCLC comparing *KRAS* mutant tumors with *KRAS* and EGFR wild-type tumors, exhibiting an insignificant trend towards increased hazard for disease recurrence after tumor resection for *KRAS* mutant patients. Neither significant heterogeneity (*I^2^*=36.0%, p=0.167) nor publication bias (Begg's test p=1.00, Egger's test p=0.334) (Figure S1B) was detected. Meta-regression analysis showed that neither race (p=0.242) nor gene mutation testing method (p=0.189) contributes significantly to the heterogeneity.

**Figure 6:**
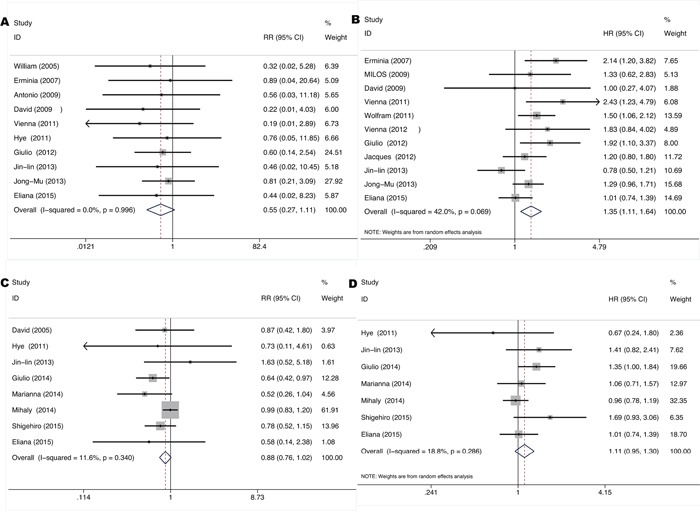
Forrest plot of relative ratio for objective response rate **A, C.** and hazard ratio of progression-free-survival **B, D.** comparing *KRAS* mutant patients with *KRAS* and *EGFR* wild-type patients treated with EGFR TKIs and chemotherapy respectively.

The total ORR to EGFR-TKIs was 2.3% (4/175) in *KRAS* mutant patients and 13.6% (101/740) in *KRAS* and *EGFR* wild-type patients based on 14 studies [9-11, 18, 19, 21, 22, 31, 32, 34, 35, 40, 42, 51] conducted in stage IIIb-IV unresectable NSCLC. As shown in Figure 6A and 6B, pooled RR was 0.55 for ORR (95% CI 0.27-1.11, p=0.095) while pooled HR was 1.35 for PFS (95% CI 1.11-1.64, p=0.002), exhibiting an insignificant trend towards lower response but significant shorter remission period of EGFR-TKIs treatment for *KRAS* mutant patients. Neither significant heterogeneity (*I^2^*=0.0%, p=0.996 and *I^2^* =42.0%, p=0.069 respectively) nor publication bias (Begg's test p=1.00, Egger's test p=0.109 and Begg's test p=0.436, Egger's test p=0.256 respectively) (Figure S1C and S1D) was detected. Meta-regression analysis showed that neither race (p=0.159) nor gene mutation testing method (p=0.801) contributes significantly to the heterogeneity.

The total ORR to chemotherapy was 35.8% (138/385) in *KRAS* mutant patients and 45.1% (381/845) in *KRAS* and *EGFR* wild-type patients based on eight studies [8, 10, 15, 27, 28, 32, 50, 51] conducted in stage IIIb-IV unresectable NSCLC. As shown in Figure 6C and 6D, pooled RR was 0.88 for ORR (95% CI 0.76-1.02, p=0.083) while pooled HR was 1.11 for PFS (95% CI 0.95-1.30, p=0.186), exhibiting an insignificant trend towards lower response and shorter remission period of chemotherapy for *KRAS* mutant patients. Neither significant heterogeneity (*I^2^*=1.6%, p=0.340 and *I^2^* =18.0%, p=0.286 respectively) nor publication bias (Begg's test p=0.902, Egger's test p=0.3 and Begg's test p=0.764, Egger's test p=0.493 respectively) (Figure S1E and S1F) was detected. Meta-regression analysis showed that race (p=0.509) doesn't contribute significantly to the heterogeneity.

### Sensitivity analyses

In general, no individual publication was found to be significantly biasing the results (Figure 2C, 2F, 3C, 3F, 4C, 4F and Figure S2A-D), but the associations between *KRAS* mutation with lower response rate and shorter remission period of chemotherapy in *EGFR* wild-type NSCLC were affected after the data set of Mihaly [28] was removed (Figure S2E, S2F). The associations shifted from statistically insignificant to significant with Mihaly et al.'s study excluded. However, this study enrolled the most patients assessed for outcomes of chemotherapy and gained a high quality score of nine stars, therefor it's unreasonable to role out this study for analyses. The sensitivity analyses showed that the cumulative results are stable.

## DISCUSSION

The *KRAS* oncogene together with *HRAS* and *NRAS* encode a family of membrane-bound 21kd guanosine triphosphate binding proteins (GTPs) that regulate cell growth, differentiation, and apoptosis by interacting with multiple signaling including mitogen-activated protein kinase (MAPK), signal transducer and activator of transcription (STAT), and phosphoinositide 3-kinase (PI3K) signaling cascades [52]. *KRAS* gene has been found frequently mutated in human tumors such as large intestine, lung and pancreas. Almost all *KRAS*-mutant cases affect exon 2 and 3 (G12, G13 and Q61), which impair the deactivation circuit of RAS proteins, thereby causing sustained activation of RAS signaling [53]. Meanwhile RAS is the most important downstream effector of EGFR, therefore sensitive mutation of *KRAS* gene might attenuate, even abolish the treatment efficacy of anti-EGFR agents such as EGFR-TKIs and EGFR monoclonal antibody (EGFR mAb). It is true that the benefit of cetuximab or panitumumab, two well-known EGFR mAb approved by FDA, is restricted to patients with *KRAS* wild-type colorectal cancer, and only this subset of patients should receive these agents [54]. Although *KRAS* is the most common mutated oncogene in NSCLC, its clinical significance is yet under debate. Should we test for it, and does it matter? Is *KRAS* testing necessary before EGFR-TKIs treatment?

The present meta-analysis with newest and largest quantity of relevant publications confirmed that *KRAS* mutation is significantly associated with worse OS (HR=1.56) and DFS (HR=1.57), and also with inferior ORR (RR=0.21 and 0.66 for TKI and chemotherapy respectively) and PFS (HR=1.46 and 1.30 respectively) of EGFR-TKIs treatment or chemotherapy, compared with *KRAS* wild-type NSCLC. While analyzing the association between *KRAS* mutation with OS, significant publication bias was detected by Egger's test (p=0.014). Thereby, a “trim and fill” method was applied. Elven hypothetical negative unpublished studies were imputed to produce a symmetrical funnel plot (Figure S3). The pooled analysis incorporating the hypothetical studies continued to show a statistically significant association between *KRAS* mutation and worse survival (HR=1.31, 95% CI 1.14-1.50 and p=0.00). Céline et al. [55] reported a significant worse survival (HR=1.35, 95% CI 1.16-1.56) of *KRAS* mutant NSCLC compared with *KRAS* wild-type NSCLC based on a meta-analysis of 28 studies early in 2004. The reported HR for OS was quit similar to ours, however, no significant survival hazard was observed in the subgroup analysis of nine studies using an immunohistochemistry (IHC) method to test RAS alternation (HR=1.08, 95% CI 0.86-1.34). Furthermore, none of the 28 studies used a direct sequencing method, which is a “gold standard” for gene testing and not spreading to clinical application until the last decade. On the contrary, none of the 41 studies included in the present meta-analysis used an IHC method. Instead, more than half of the included studies used a direct sequencing method. As more included studies, more enrolled cases and more developed gene testing method, our results are more reliable.

Resistance to EGFR-TKIs treatment for *KRAS* mutant NSCLC was also reported in other two meta-analysis conducted by Chen et al. [56] and Min et al. [57]. The reported pooled RR for ORR was 0.29 (95% CI 0.18-0.47) in Chen's study and 0.21 (95% CI 0.12-0.39) in ours while the reported pooled HR for PFS was 1.86 (95% CI 1.51-2.29) in Min's study and 1.46 (95% CI 1.23-1.74) in ours, showing highly consistent results among studies. Meanwhile, the present meta-analysis included more publications and presented more accurate confidence interval. Resistance to chemotherapy for *KRAS* mutant NSCLC was also reported by another meta-analysis [58]. The reported odds ratio (OR) was 0.67 (95% CI 0.50-0.88) for ORR with statistical significance and 0.75 (95% CI 0.54-1.04) for 6 month and 1-year PFS rate but without statistical significance. Only first-line chemotherapy was evaluated. We doubt that HR might be more suitable than OR for analyzing PFS, which displayed an abnormal distribution. Our results showed both significant inferior ORR (RR=0.67, 95% CI 0.50-0.88) and PFS (HR=1.30, 95% CI 1.14-1.50) for the *KRAS* mutant patients.

Additionally, we noticed that *KRAS* mutation impairs OS and DFS more obviously in the Asians (HR=2.39 and 2.59 respectively) than in the Caucasians (HR=1.37 and 1.31 respectively), which is not reported elsewhere. It is believed that *KRAS* mutation subtypes have diverse prognosis and respond differently to chemotherapy or EGFR-TKIs [15, 25, 28, 47, 48, 59]. The author speculated that different spectrum of *KRAS* mutation subtypes, especially increased proportion of G13, G12D and G12V in the Asians, might be partly responsible for the different hazard ratio between two races. Secondly, there were more *KRAS* wild-type cases than *KRAS* mutant cases enrolled in studies. This unbalanced situation was more obviously in studies conducted in Asia, which might exaggerate the HRs for OS and DFS in the Asians. More detailed mechanisms need to be exploited in future fundamental research focused on divergence of RAS signal transduction between two races.

Besides *KRAS*, oncogene *EGFR* is also frequently mutated in NSCLC, which predicts dramatic benefits from EGFR-TKIs treatment [3, 8, 17–20, 23, 31], and even from chemotherapy [32, 49]. Mutations of *KRAS* and *EGFR* are generally mutually exclusive in NSCLC, i.e. most *EGFR* mutations were existed in *KRAS* wild-type patients, which might bias the results toward an overestimation of the prognostic and predictive value of *KAS* mutation. Thus, we carried out further analyses in *EGFR* wild-type NSCLC to draw a more objective conclusion of clinical significance of *KRAS* mutation. While compared with *KRAS* and *EGFR* wild-type NSCLC, the prognostic and predictive value of *KRAS* mutation did decreased. Pooled HR decreased from 1.56 and 1.57 to 1.40 and 1.33 for OS and DFS respectively, yet statistically significant for OS (p=0.0) but not for DFS (p=0.076). Similarly, *KRAS* mutation impaired OS and DFS (without statistical significance, data not shown) more seriously in the Asians. Pooled RR for ORR increased from 0.21 and 0.66 to 0.55 and 0.88 for EGFR-TKIs treatment and chemotherapy respectively. No statistical significances were observed (p=0.095 and 0.813 respectively). Pooled HR for PFS decreased from 1.46 and 1.30 to 1.35 and 1.11 for EGFR-TKIs treatment and chemotherapy respectively. Statistical significance was observed in EGFR-TKIs treatment (p=0.002), but not in chemotherapy (p=0.186). Although associations of *KRAS* mutation with inferior treatment outcomes turned out to be statistically insignificant, the results seemed unstable. Sensitivity analyses showed that the associations of *KRAS* mutation with inferior chemotherapy outcomes were significantly affected after Mihaly et al.'s study was removed. It is noteworthy that there were fewer studies evaluating the associations of *KRAS* mutation with treatment outcomes in *EGFR* wild-type NSCLC. Besides, obvious trends towards inferior treatment outcomes and borderline confidence intervals were observed, the author speculated that *KRAS* mutation is still a valid predictor for poor treatment outcomes in *EGFR* wild-type NSCLC with more publications to be included. However, its prognostic and predictive value is not so remarkable as it was greatly affected by exclusion of *EGFR* mutant patients and the HRs for OS, DFS and PFS were no more than two fold. Actually only NSCLC patients with sensitive *EGFR* mutation are recommend to first line EGFR-TKIs treatment according to NCCN Guidelines. Based on the notion that mutations of *EGFR* and *KRAS* are generally mutually exclusive, a very few *KRAS* mutant patients are subjected to EGFR-TKIs treatment. Therefore *KRAS* testing is of limited value to optimize the use of EGFR-TKIs in clinic compared to *EGFR* testing.

Despite our efforts in performing a comprehensive and accurate analysis, yet several limitations should be taken into consideration when interpreting the findings. Firstly, fewer studies assed the predictive and prognostic value of *KRAS* mutation in *EGFR* wild-type NSCLC. Thus borderline significant associations of *KRAS* mutation with inferior treatment outcomes were observed. Secondly, the present study is a univariate analysis. Although several factors such as race, stage, gene testing method and *EGFR* mutational status were taken into consideration, other factors such as *KRAS* mutation subtypes, other gene mutational status as *ALK* rearrangement [10, 15] and *PIK3CA* mutation [21, 42, 46, 60], performance status and smoking status should not be neglected in the analysis with more available data provided in the future studies. Lastly, it is noteworthy that *KRAS* mutation, and even subtype-specific *KRAS* mutations, responds differently to different chemotherapeutics [34, 61]. Therefore, associations between subtype-specific *KRAS* mutations and responses to specific chemotherapeutics should be strictly exploited in future studies.

In conclusion, *KRAS* mutation is a weak, but valid predictor for poor prognosis and treatment outcomes for surgical resection, EGFR-TKIs treatment or chemotherapy. Its prognostic and predictive value is greatly impaired when *EGFR* mutant patients were excluded. One thing for sure is that it closely related to a worse survival irrespective of *EGFR* mutational status especially for the Asians. So far, no effective treatment method direct targeting mutant *KRAS* gene has been approved in clinic. Agents interrupting RAS signaling such as *MEK* inhibitor [62–64] or miR-126 [65] seemed selective effective for *KRAS* mutant tumors, which could be utilized for the development of target therapy for *KRAS* mutant tumors and might overcome the survival hazard induced by KRAS mutation.

## MATERIALS AND METHODS

### Publication search and selection

The identification of potential relevant studies was performed through a systemic search in PubMed, Embase and Web of Science databases using the following keywords “lung cancer”, “non-small cell lung cancer” or “NSCLC” and “*KRAS*”. The latest search was updated on September 2015. Bibliographies of eligible studies, review articles and other relevant publications were also reviewed to identify all potential studies.

A study had to fulfill the following criteria: (1) to deal with non-small cell lung cancer (any stage); (2) to stratified by *KRAS* mutational status; (3) to assess the correlation between *KRAS* mutation and survival or treatment outcome (surgery, EGFR-TKIs, platinum-based chemotherapy); (4) to have been published as a full paper in the English language and in the last ten years (2005-2015). The studies were excluded from the analysis if any of the cases occurred: (a) EGFR-TKIs and platinum-based chemotherapy were used as neo-adjuvant treatment; (b) critical information was missing or could not be obtained by our repeated quests.

### Data extraction

Two investigators (Wei Pan and Yan Yang) independently screened the studies and extracted the data from included studies by using standard data-abstraction forms. Disagreements were resolved through discussion with another investigator (Hongcheng Zhu). For each study, the following characteristics and information were collected: first author, year of publication, number of patients assessed for *KRAS* gene and number of patients bearing *KRAS* mutation gene, gene mutation detection method, ethnicity, pathology, clinical stage and data linking *KRAS* mutation to treatment outcomes (i.e., CR+PR, SD, PD, and PFS). If a direct report of HR and 95% CI was not available, the total number of events, the number of patients at risk in each group and the log-rank statistic or its P-value was used to allow for an approximation of the HR estimate. If above parameters were yet unavailable, estimated value was derived indirectly from Kaplan-Meier curves using the methods described by Tierney et al. [66]. Survival rates on Kaplan-Meier curves were read by Engauge Digitizer version 4.1 (http://digitizer.sourceforge.net), and then the data read from the curves were entered in the calculation spreadsheet appended to Tierney's paper.

### Statistical methods

We extracted relative risks (RRs) with its 95%CIs to show the strength of the association between *KRAS* mutation and objective response rate (CR + PR), and hazard ratios (HRs) with its 95%CIs to show the survival (OS, DFS or PFS) benefits of *KRAS* mutant tumors. The individual RRs and HRs were combined into pooled RR and HR, and the initial analyses were performed with a fixed effect model assuming homogeneity of the individual studies. Heterogeneity assumption was checked by Q-test and *I*^2^ test. A significant Q-test (*p*<0.05) or *I*^2^>50% indicate the heterogeneity among the studies, and the random-effect model was applied for meta-analysis.

Meta-regression analyses were generated to explore possible sources of heterogeneity (adjusted R^2^>50% and p<0.05 were consider significant).

Sensitivity analyses were conducted to identify whether results of the meta-analysis were signify affected by exclusion of any individual study and to testify the reliability of the conclusions.

Begg's and Egger's tests were used to evaluate the potential publication bias. The tests were considered statistically significant if p<0.05, and a non-parametric “trim-and-fill” method was applied. All *p* values were 2-sided and all analyses were performed using Stata SE 11.0 software.

## SUPPLEMENTARY FIGURES AND TABLES




