# Supplementary material for: KRAS mutation is a weak, but valid predictor for poor prognosis and treatment outcomes in NSCLC: A meta-analysis of 41 studies
Source: Oncotarget. 2016 Jan 30;7(7):8373–88. doi: 10.18632/oncotarget.7080 (PMC4884999; doi:10.18632/oncotarget.7080)
Supplement: Supplementary file 1 [file oncotarget-07-8373-s001.pdf]

SUPPLEMENTARY FIGURES AND TABLES

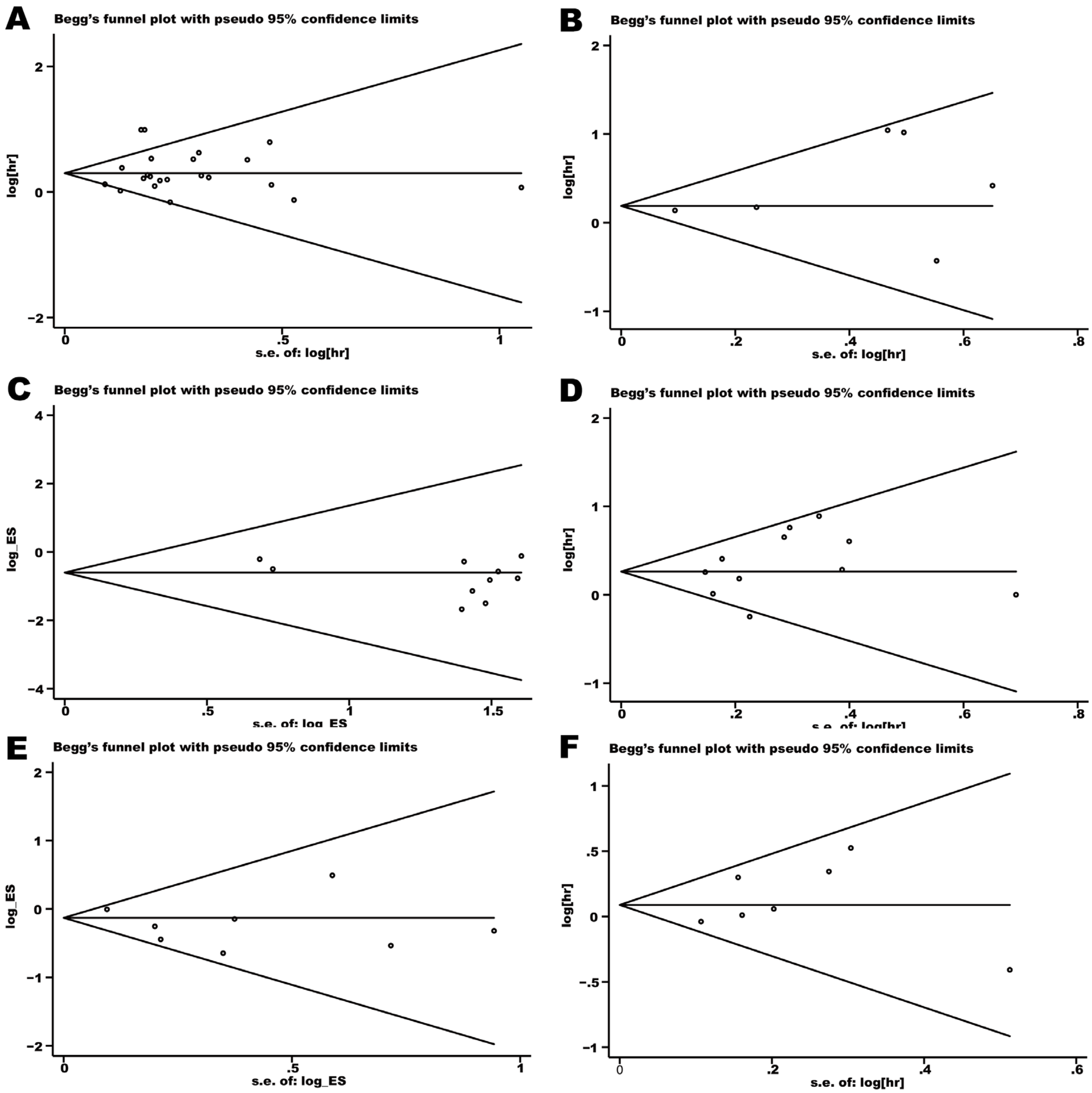

Supplementary Figure S1: Begg's funnel plot of enrolled studies for estimating hazard ratio for overall survival **A.,** disease-free-survival **B.** and progression-free-survival (**D.** for TKIs, **F.** for chemotherapy), and for estimating relative ratio for objective response rate (**C.** for TKIs, **E.** for chemotherapy) comparing *KRAS* mutant patients with *KRAS* and *EGFR* mutant patients.

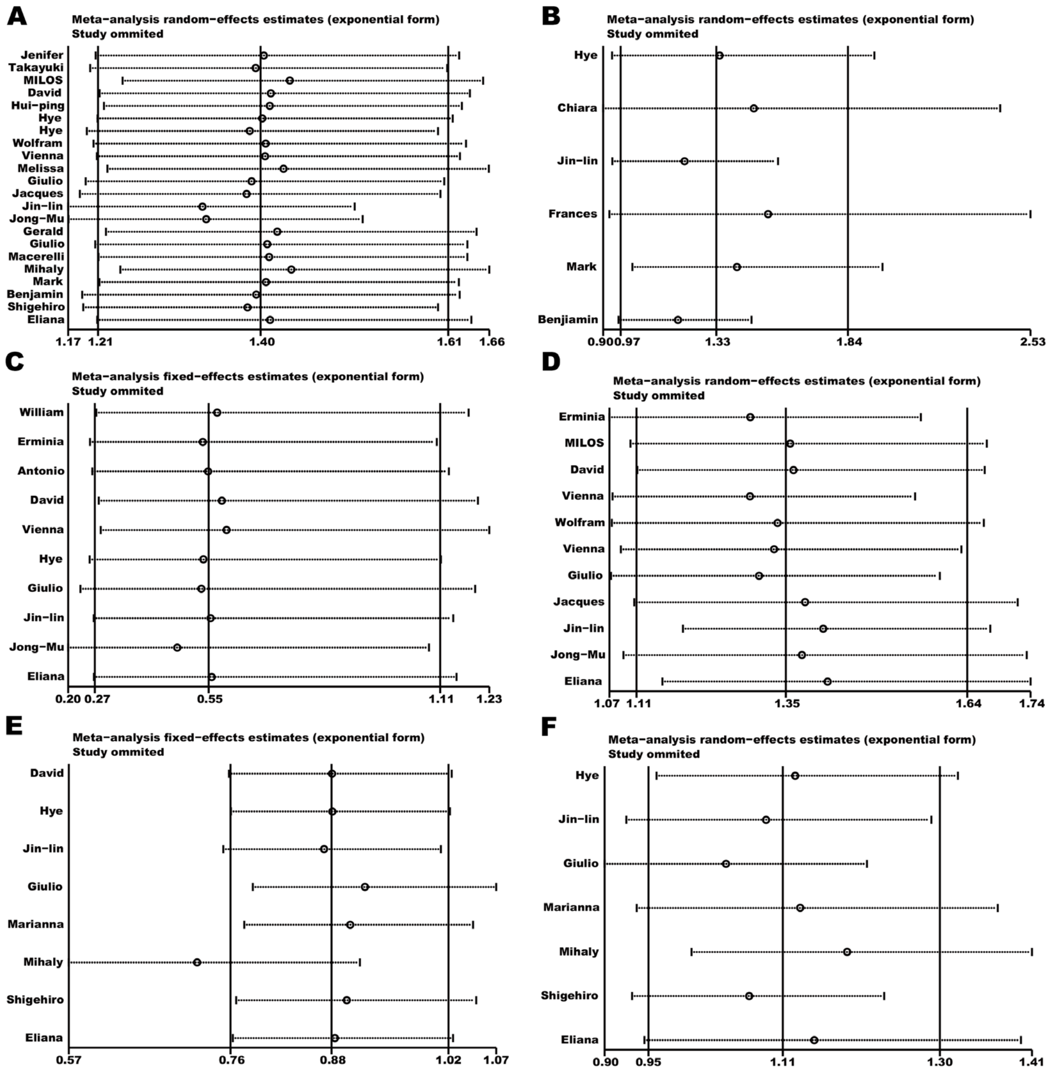

Supplementary Figure S2: Influence analysis of hazard ratio for overall survival **A.**, disease-free-survival **B.** and progression-free-survival (**D.** for TKIs, F for chemotherapy), and of relative ratio for objective response rate (**C.** for TKIs, **E.** for chemotherapy) comparing *KRAS* mutant patients with *KRAS* and *EGFR* mutant patients.

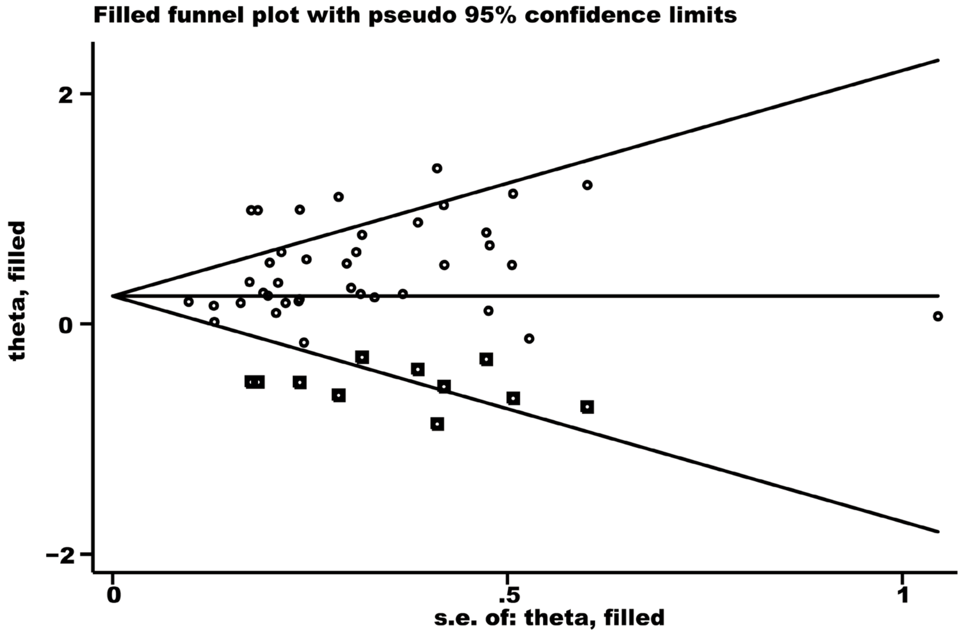

Supplementary Figure S3: Begg's funnel plot of enrolled studies and elven hypothetical negative unpublished studies for estimating hazard ratio for overall survival comparing *KRAS* mutant patients with *KRAS* wild-type patients.

Supplementary Table S1: Detailed quality score of included studies

See Supplementary File 1

Supplementary Table S2: Meta-analysis on the association between demographic characters and *KRAS* mutations

	KRAS MUT	*KRAS* WT	Test of association			Test of heterogeneity	
			RR	95% CI	*P*	*P*	I^2 (%)
Gender							
Male	773	2331	1.07	0.97-1.18	0.158	0.00	64.2
Female	646	1880					
Smoking history							
Former or current	1146	2656	1.16	1.08-1.25	0.00	0.00	78.8
Never	196	1145					
Histology							
ADC	488	1489	1.13	1.03-1.24	0.01	0.00	71.0%
Other	112	557					

MUT, mutation**; WT,** wild-type**; RR,** relative ratio**; ADC,** lung adenocarcinoma.
