# Supplementary material for: KRAS mutation is a weak, but valid predictor for poor prognosis and treatment outcomes in NSCLC: A meta-analysis of 41 studies
Source: Oncotarget. 2016 Jan 30;7(7):8373–88. doi: 10.18632/oncotarget.7080 (PMC4884999; doi:10.18632/oncotarget.7080)
Supplement: Supplementary file 2 [file oncotarget-07-8373-s002.docx]

**Table S1: Detailed quality score of included studies**

| First Author | Selection | | | | Comparability | Outcome | | | Total Score |
| --- | --- | --- | --- | --- | --- | --- | --- | --- | --- |
|  | **Representative of the exposed cohort** | **Selection of the non exposed cohort** | **Ascertainment of exposure** | **Outcome was not present at start of study** | **Based on the design or analysis** | **Assessment of outcome** | **Follow-up long enough** | **Adequacy of follow-up** |  |
| William [35] | 0 | 1 | 1 | 1 | 0 | 1 | 1 | 1 | 6 |
| David [8] | 1 | 1 | 1 | 1 | 1 | 1 | 1 | 1 | 8 |
| Erminia [9] | 0 | 1 | 1 | 1 | 2 | 1 | 1 | 1 | 8 |
| Oliver [38] | 1 | 1 | 0 | 1 | 1 | 1 | 1 | 1 | 6 |
| Young [29] | 0 | 1 | 1 | 1 | 0 | 1 | 1 | 1 | 6 |
| Jenifer [16] | 1 | 1 | 1 | 1 | 2 | 1 | 1 | 1 | 9 |
| Chang-qi [17] | 1 | 1 | 1 | 1 | 2 | 1 | 1 | 1 | 9 |
| Takayuki [39] | 1 | 1 | 1 | 1 | 2 | 1 | 1 | 1 | 9 |
| MILOS [18] | 0 | 1 | 1 | 1 | 1 | 1 | 1 | 1 | 7 |
| David [19] | 1 | 1 | 1 | 1 | 1 | 1 | 1 | 1 | 8 |
| Tetsukan [30] | 1 | 1 | 1 | 1 | 2 | 1 | 1 | 1 | 9 |
| Antonio [40] | 0 | 1 | 1 | 1 | 1 | 1 | 1 | 1 | 7 |
| Hui-ping [20] | 0 | 1 | 1 | 1 | 2 | 1 | 0 | 1 | 7 |
| Laura [41] | 0 | 1 | 1 | 1 | 2 | 1 | 1 | 1 | 8 |
| Vienna [21] | 0 | 1 | 1 | 1 | 2 | 1 | 1 | 1 | 8 |
| Carlos [12] | 1 | 1 | 0 | 1 | 2 | 1 | 1 | 1 | 8 |
| Hye [10] | 0 | 1 | 1 | 1 | 0 | 1 | 1 | 1 | 6 |
| Wolfram [22] | 1 | 1 | 1 | 1 | 2 | 1 | 1 | 1 | 9 |
| Vienna [42] | 1 | 1 | 1 | 1 | 2 | 1 | 1 | 1 | 9 |
| Chiara [43] | 1 | 1 | 1 | 1 | 2 | 1 | 1 | 1 | 9 |
| Melissa [23] | 1 | 1 | 1 | 1 | 2 | 1 | 1 | 1 | 9 |
| Jie [44] | 0 | 1 | 1 | 1 | 0 | 1 | 1 | 1 | 6 |
| Giulio [11] | 0 | 1 | 1 | 1 | 1 | 1 | 1 | 1 | 7 |
| Jacques [31] | 0 | 1 | 1 | 1 | 1 | 1 | 1 | 1 | 7 |
| Seung [45] | 0 | 1 | 1 | 1 | 2 | 1 | 1 | 1 | 8 |
| Anneli [13] | 1 | 1 | 1 | 1 | 1 | 1 | 1 | 1 | 8 |
| Ondrej [46] | 0 | 1 | 1 | 1 | 1 | 1 | 1 | 1 | 7 |
| Jong-Mu [34] | 1 | 1 | 1 | 1 | 2 | 1 | 1 | 1 | 9 |
| Frances [25] | 1 | 1 | 1 | 1 | 2 | 1 | 1 | 1 | 9 |
| Gerald [26] | 0 | 1 | 1 | 1 | 1 | 1 | 1 | 0 | 6 |
| Wouter [14] | 0 | 1 | 1 | 1 | 2 | 1 | 1 | 1 | 8 |
| Giulio [15] | 1 | 1 | 1 | 1 | 2 | 1 | 1 | 1 | 9 |
| Marianna [27] | 1 | 1 | 1 | 1 | 2 | 1 | 1 | 1 | 9 |
| Mihaly [28] | 1 | 1 | 1 | 1 | 2 | 1 | 1 | 1 | 9 |
| Mark [24] | 1 | 1 | 1 | 1 | 1 | 1 | 1 | 1 | 8 |
| Benjamin [47] | 0 | 1 | 1 | 1 | 2 | 1 | 1 | 1 | 8 |
| Ernest [48] | 0 | 1 | 1 | 1 | 2 | 1 | 1 | 1 | 8 |
| Alma [49] | 1 | 1 | 1 | 1 | 1 | 1 | 1 | 1 | 8 |
| Shigehiro [50] | 0 | 1 | 1 | 1 | 2 | 1 | 1 | 1 | 8 |
| Eliana [51] | 0 | 1 | 1 | 1 | 2 | 1 | 1 | 1 | 8 |

**Evaluation based on NOS Scale**

| First Author | Randomization | Double blinding | Withdrawals and dropouts | Total |
| --- | --- | --- | --- | --- |
| Jin-lin[32] | 1 | 1 | 1 | 3 |

**Evaluation based on Jadad Scale**
